# Return to Sport After Shoulder Injuries in Mixed Martial Arts: Implications on Longevity and Performance

**DOI:** 10.3390/jcm14113767

**Published:** 2025-05-28

**Authors:** Mohamad Y. Fares, Ryan Stadler, Jack Mao, Diane Ghanem, Peter Boufadel, Mohammad Daher, Tarishi Parmar, Evangeline F. Kobayashi, Adam Z. Khan, Hafiz F. Kassam, Joseph A. Abboud

**Affiliations:** 1 Division of Shoulder and Elbow Surgery, Rothman Orthopaedic Institute, Philadelphia, PA 19107, USA; paboufadel@gmail.com (P.B.); mohdaher1@hotmail.com (M.D.); tarishiparmar20051@gmail.com (T.P.); fuminakobayashi@gmail.com (E.F.K.); abboudj@gmail.com (J.A.A.); 2 Rutgers Robert Wood Johnson Medical School, New Brunswick, NJ 08901, USA; rds244@rwjms.rutgers.edu; 3 School of Medicine, Wayne State University, Detroit, MI 48201, USA; hn1730@wayne.edu; 4 Department of Orthopaedic Surgery, Johns Hopkins University, Baltimore, MD 21287, USA; diane.ghanem@gmail.com; 5 Southern Permanente Medical Group, Pasadena, CA 90034, USA; 6 Newport Orthopedic Institute, Newport Beach, CA 92660, USA; hafizkassam@googlemail.com

**Keywords:** MMA, labral tear, knockdown rate, takedown rate, fighter rehabilitation

## Abstract

**Background/Objectives:** Mixed martial arts (MMA) is a combat sport which heavily involves upper limb strength, mobility, and stability. Shoulder injuries, given their impact on striking and grappling, may significantly hinder performance and career longevity. However, their specific effects on competitive outcomes remain poorly defined. This study evaluates return-to-sport rates, fight performance, and long-term success in professional MMA athletes following shoulder injuries. **Methods**: A retrospective cohort study was conducted using publicly available databases to identify professional MMA fighters from the UFC, Bellator, and Strikeforce who sustained shoulder injuries requiring withdrawal from scheduled bouts. Fighter demographics, injury characteristics, and treatment approaches were recorded. Performance metrics—including winning percentage, takedown (TD), knockdown (KD), and significant strike (SS) rates—were compared before and after injury. Independent *t*-tests were used, and significance was set at *p* < 0.05. **Results**: A total of 27 fighters with 34 documented shoulder injuries were included. The most common injury was a torn labrum (41.2%), with 76.5% requiring surgical intervention. Aggregate winning rates significantly declined from 81.96% pre-injury to 54.7% post-injury (*p* < 0.001). Aggregate KD rates also dropped significantly (*p* < 0.001), while TD rates trended downward without reaching statistical significance. SS rates remained stable, suggesting potential compensatory mechanisms. Injury recurrence was observed in 22.2% of cases. **Conclusions**: Shoulder injuries in MMA are associated with a substantial decline in competitive success, particularly in knockout capability, emphasizing the critical role of shoulder integrity in fight performance. The high recurrence rate suggests the need for optimized rehabilitation protocols and stricter return-to-sport guidelines to enhance fighter longevity.

## 1. Introduction

Mixed martial arts (MMA) is a rapidly growing combat sport within the United States [1,2]. Due to its inherently high-contact nature, MMA carries a high risk of injury, with reported rates as high as 51 injuries per 100 athlete exposures [3]. Though several studies have sought to characterize MMA injury patterns, these often tend to focus on the epidemiology of these injuries, with a main interest in head and neck trauma [4,5,6]. The literature remains scarce regarding other types of frequently encountered injuries and their sequelae, particularly those targeting the upper limb. In 2022, Fares et al. examined post-fight ringside physician reports to describe upper-limb injury patterns in the Ultimate Fighting Championship (UFC) [7]. The authors looked into the profile of all upper-limb injuries in the sport and noted a higher incidence of hand injuries when compared to other anatomic locations, while still noting a significant number of injuries affecting the shoulder region [7]. Given the importance of the shoulder in executing striking and grappling techniques, a shoulder injury in MMA can lead to devastating outcomes for the competing fighter [7].

A higher degree of shoulder mobility, strength, and stability can have an immense impact in MMA, and these parameters can lead to significant differences in strike force and reach [8,9]. That being said, the joint’s extensive range of motion and dynamic stability renders it particularly vulnerable to injury, especially in a sport like MMA, where high-velocity impacts and forceful submissions are routine [7]. Additionally, ligamentous shoulder injuries, such as dislocations and subluxations, can lead to recurrent instability, often necessitating surgical intervention and entailing prolonged recovery periods for the injured athlete [7,10,11,12]. For those who have endured years of training to compete at the highest level, failing to return from injury is not only distressing but may even result in professional and economic losses [13].

Although return-to-sport outcomes following shoulder injuries have been examined in baseball, football, and soccer players, no study to date has evaluated the impact of these injuries on the performance of professional MMA fighters returning to action in the Ultimate Fighting Championship (UFC) [14,15,16]. Given the involvement of the joint in the different aspects of fighting, as well as the financial and career implications of injury-related performance decline, it is important to assess how shoulder injuries affect competitive success in this unique athletic population. The present study aims to evaluate return-to-sport rates, fight performance, and long-terms outcomes in professional MMA fighters following shoulder injuries.

## 2. Materials and Methods

### 2.1. Study Design

This is a retrospective cohort study that evaluated professional MMA athletes’ ability to return to sport after shoulder injury and their performance prior to and after injury/treatment. Data were collected for all injured athletes in major fighting promotions, which include the UFC, Bellator, and Strikeforce. Inclusion criteria consisted of all athletes who suffered a reported shoulder injury either during training or competition that required them to withdraw from a scheduled bout. Our study is retrospective in nature, and it utilizes data from publicly available resources; as such, an institutional review board was not required.

### 2.2. Data Collection

Three publicly accessible prominent MMA news media outlets (sherdog.com, bloodyelbow.com, and mmafighting.com, accessed on 15 August 2024) were used to conduct an initial screen of athletes who suffered shoulder injuries, since the inception of the databases and up until 1 October 2024 [17,18,19]. The search terms “shoulder”, “shoulder injury”, and “shoulder surgery” were the keywords used for this search. Data pertaining to the shoulder injury sustained were collected, including injury date, setting (training vs. competition), and type, if disclosed [17,18,19]. If the data pertaining to a specific variable was not reported, it was labeled in our database as “unknown”.

To obtain additional fighter-specific data, official UFC and Bellator websites were screened [20,21]. Information collected included fighter demographics (age, gender, weight class), fight records, and match results [20]. Return to sport dates were recorded and the outcomes of interest were the immediate and aggregate winning rates of athletes prior to and after a reported shoulder injury. Immediate winning rates were defined as an athlete’s win rate two fights before and after an injury, and aggregate rates were defined as an athlete’s total win rate before and after injury. Additional parameters like number of takedowns (TD), knockdowns (KD), and significant strikes (SS) for all the competitions an athlete participated in within in the organization were also recorded [20,21]. Statistics involving fight parameters (TDs, KDs, and SS rates) were adjusted to a per-minute basis to account for different fight lengths.

### 2.3. Statistical Analysis

An independent *t*-test was used to compare between the immediate and aggregate winning rates before and after the reported shoulder injury. This test was also used to compare between the rate of TDs, KDs, and SSs before and after the reported shoulder injuries. Statistical analysis was performed using the Statistical Package for the Social Sciences software (Version 26.0.0). A *p* value less than 0.05 was considered statistically significant.

## 3. Results

### 3.1. Fighter Demographics

A total of 27 athletes were included in this study. All included fighters were male. The average age at injury was 32.84 years (Range: 22–44). The majority of fighters (92.6%) were affiliated with the UFC, and only two fighters were affiliated with the organizations Bellator and Strikeforce. Recurrent shoulder injuries were reported in six fighters (22.2%).

Athletes from various weight classes were represented, with the heavyweight division being the most common, with five injured fighters (18.5%), followed by flyweight and light heavyweight classes, with four injured fighters each (14.8%). Bantamweight, featherweight, welterweight, and middleweight classes each had three injured athletes (11.1% each). The fewest number of injuries were seen among fighters in the lightweight class, with only two injured athletes (7.4%). Overall, fighters were more likely to experience a unilateral shoulder injury (81.5%) compared to bilateral injuries (18.5%). Table 1 summarizes the demographic characteristics of the study cohort.

### 3.2. Injury Characteristics

**Figure 1:**
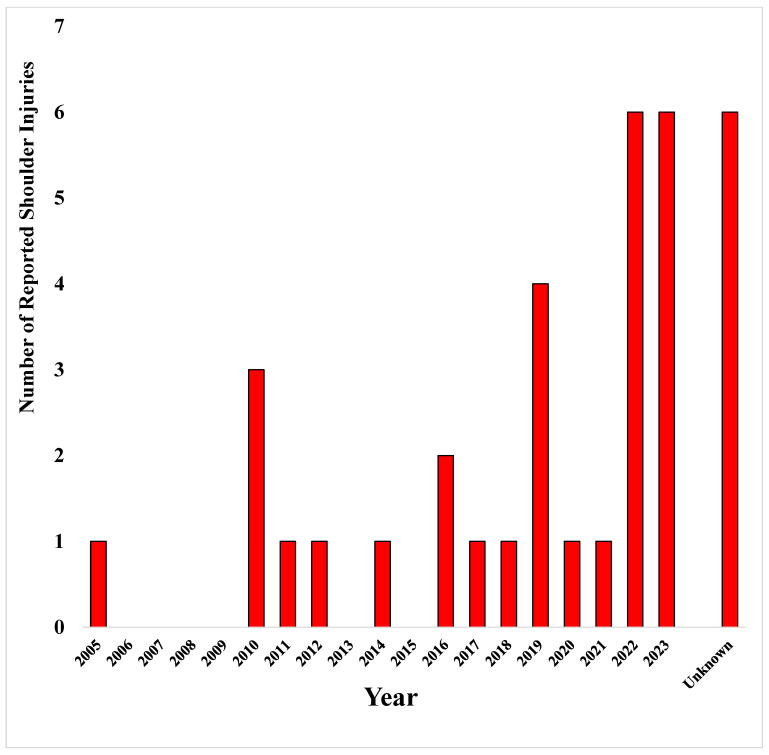
Distribution of reported shoulder injuries across the years.

A total of 34 shoulder injuries were identified among the 27 athletes, as some fighters sustained more than one injury. The highest number of reported shoulder injuries occurred in 2022 and 2023, with six cases each (Figure 1).

The most common shoulder injury type was an isolated labral tear, which occurred 14 times. There were four incidences of a torn labrum with concurrent tear of the rotator cuff, three incidences of a rotator cuff tear injury, and three incidences of AC joint tears. Combined labrum, rotator cuff, and biceps tear injuries occurred twice, as did injuries to the long head of biceps complex and SLAP tears. Six injuries had an unknown shoulder injury type (Table 2).

A total of 26 injuries required surgical intervention, five injuries were treated conservatively, and the management of three injuries was unable to be obtained. The setting where the injury took place was fairly evenly distributed, with fifteen injuries occurring during a professional fight and thirteen injuries occurring during training. Six injuries had no information about their setting. Table 2 shows the different injury characteristics of the included fighters in our study.

### 3.3. Changes in Winning Rates Following Injuries

Of the 34 injuries, three fighters did not compete after injury, leaving 31 shoulder injury incidents for analysis.

Immediately post-injury, 14 shoulder injuries (45.2%) resulted in a decreased winning rate, 11 injuries (35.5%) showed no change in winning rate, and 6 injuries saw an increase in winning rate. The mean immediate pre-injury winning rate was 77.4%, which declined to 60.35% post-injury, albeit without reaching significance (*p* = 0.073). Aggregately post-injury, 26 injuries (83.9%) resulted in a decreased winning rate, 1 injury (3.2%) had no change in winning rate, and only 4 injuries saw an increase in winning rate. The mean aggregate pre-injury winning rate was 81.96%, which significantly declined to 54.7% post-injury (*p* < 0.001).

### 3.4. Changes in Fight Parameters

Two fighters, sustaining one shoulder injury each, did not have fight parameter records prior to or after their injury. As such, a total of 29 shoulder injuries were included for pre- and post-injury fight parameter analysis (TD, KD, and SS rates).

Immediately following shoulder injury, a lower takedown rate was seen in 14 cases, a greater takedown rate was seen in 9 cases, and 6 cases saw no change in takedown rate. Similarly, a lower knockdown rate was seen in 16 cases, a greater knockdown rate was seen in 7 cases, and 6 cases saw no change in knockdown rate. Finally, a lower rate of significant strikes was seen in 18 cases, and a greater rate of significant strikes was seen in 11 cases.

Aggregately following a shoulder injury, a lower takedown rate was seen in 21 cases, while a greater takedown rate was seen in 8 cases. Similarly, a lower knockdown rate was seen in 26 cases, while a greater knockdown rate was seen in 3 cases. Finally, a lower rate of significant strikes was seen in 16 cases, and a greater rate of significant strikes was seen in 13 cases.

The mean immediate pre-injury takedown rate was 0.19 takedowns per minute, greater than the mean post-injury takedown rate of 0.13 takedowns per minute, albeit without reaching significance (*p* = 0.403). Similarly, the mean aggregate pre-injury takedown rate post shoulder injury was 0.16 takedowns per minute, greater than the mean post-injury takedown rate of 0.12 takedowns per minute, without reaching significance (*p* = 0.297).

The mean immediate pre-injury knockdown rate was 0.1 knockdowns per minute, greater than the mean post-injury knockdown rate of 0.03 takedowns per minute, albeit without reaching significance (*p* = 0.114). The mean aggregate pre-injury knockdown rate was 0.05 knockdown per minute, significantly greater than the mean post-injury knockdown rate of 0.017 knockdowns per minute (*p* < 0.001).

The mean immediate pre-injury rate of significant strikes was 4.7 significant strikes per minute, greater than the mean post-injury rate of significant strikes of 3.97 significant strikes per minute, albeit without reaching significance (*p* = 0.356). However, the mean aggregate pre-injury rate of significant strikes was 4.04 significant strikes per minute, less than the mean post-injury rate of significant strikes of 4.34 significant strikes per minute, without reaching significance (*p* = 0.748).

## 4. Discussion

Our study provided valuable insights regarding the impact of shoulder injuries on the performance of MMA athletes. A large portion of the documented shoulder injuries were seen in 2022 and 2023, most likely due to the better reporting by media outlets and the growing popularity of the sport. Different shoulder injury types were seen among different weight divisions and in both training and competitive settings in our study. Labral tears were the most commonly encountered injury type, emphasizing the critical role of the glenohumeral stability in MMA. Our data show that shoulder injuries led to significant declines in both aggregate winning and KD rates, underscoring their detrimental effects on competitive success. While TD rates and immediate post-injury winning rates also exhibited downward trends, these changes did not reach statistical significance, suggesting that the long-term repercussions of shoulder injuries may be more pronounced than their immediate effects. These results showcase the potentially detrimental effects of shoulder injuries on the longevity and performance of MMA athletes.

Both immediate and aggregate winning rates witnessed declines following shoulder injuries, albeit only the decline in aggregate winning rates was deemed significant on analysis. Several factors can attribute to this finding. Shoulder injuries, especially those that require surgery, often necessitate lengthy periods for rehabilitation and recovery [14,15,16,22,23]. As such, this leads to a decline in overall fight-readiness and physical conditioning [14,15,16,22,23]. Moreover, the shoulder joint is heavily implicated in striking and grappling techniques in MMA, and any injury can compromise function and hinder performance [7]. Labral tears were the most commonly encountered shoulder injury type in our study, suggesting that glenohumeral instability may be particularly concerning in impacting athletic performance, as documented by other contact sports in the literature [12,24,25,26].

In this setting, however, it is important to note that confounding factors may play a role in impacting aggregate winning rates. MMA fighters often face easier competition early on in their careers and are expected to face tougher competition as they progress in their careers. That, along with naturally declining athletic performance due to aging, may have also contributed to the observed decrease in aggregate winning rates [9]. That being said, the fact that the mean immediate winning rate pre-injury (77.4%) was similar to the aggregate winning rate pre-injury (81.96) suggests that these confounding variables may not have played a significant role in our analysis.

When exploring fight parameters, our study showed a trend towards decreased rates of takedowns and knockdowns following shoulder injuries. Although the decline in takedown rates did not reach statistical significance, the decline in aggregate knockdown rates was deemed statistically significant (*p* < 0.001). This finding suggests that shoulder injuries can affect the ability to deliver fight-ending strikes more so than the ability to initiate grappling techniques, which is particularly relevant, given how achieving KO/TKO finishes is often considered a significant determinant of success in MMA [4,27,28,29]. Interestingly though, the rate of significant strikes did not follow the same trend as that of other fight parameters. In fact, the aggregate rate of significant strikes post-injury (4.34) was slightly higher than that pre-injury (4.04 per minute). Given the importance of upper-limb strength in striking, fighters may compensate for reduced power post-injury by increasing strike frequency or relying more on lower-limb striking techniques.

Shoulder injuries occurred in both training and competitive settings, reflecting the intense training practices in the sport and the multifaceted injury risks associated with its participation [5]. Injuries can occur due to high-impact exchanges or submission maneuvers exhibited in competition or in high-intensity sparring, overuse due to repetitive stress, or the improper execution of techniques and drills [2,5,7,28,30]. This highlights the roles of trainers and coaches in appropriately managing training sessions to avoid fatigue, prevent aggressive sparring, highlight technical adherence, and ensure fighter safety.

Around 22.2% of shoulder injuries showed recurrence, and around 76.5% required surgical intervention. These high recurrence and surgical rates entail significant periods of rehabilitation, preventing the fighter from competing for lengthy periods. This, in turn, can demonstrate some of the challenges associated with shoulder injuries in MMA. The dynamic nature of MMA, along with the significant professional stresses that necessitate frequent participation in fights, may compromise proper post-injury rehabilitation and entail a premature return to competition [5,27,31]. As a result, this may lead to increased rates of re-injury and recurrence. Our study aligns with numerous studies in the literature that demonstrated a recurrence of instability in contact athletes after shoulder instability events [16,31,32,33]. As such, several recommendations can be derived in hopes of achieving proficient return-to-sport policies, ensuring fighter safety and longevity in the sport of MMA.

From a practical standpoint, trainers and therapists working with MMA fighters should implement athlete-tailored rehabilitation protocols following shoulder injury, with a major focus on restoring stability and sport-specific functions to the glenohumeral joint. This can be achieved by strengthening the dynamic stabilizers of the joint and emphasizing neuromuscular control techniques that mitigate the risk of recurrent injury [34]. In addition, MMA organizations should implement injury surveillance and rehabilitation policies that allow for the confidential identification of injured fighters, in both competitive and training settings, and emphasize appropriate medical clearance regulations that ensure optimal recovery prior to return to sport and competition. Incentivizing and compensating injured fighters under contract in this setting is key to ensure compliance and adherence. Additionally, publishing deidentified yet detailed analyses of these injuries can be very helpful in extrapolating effective preventive and therapeutic strategies in the future.

Our study is the first to explore return to sport following shoulder injuries in the sport of MMA. That being said, several limitations exist. Our study was limited by its retrospective nature and the reliance on publicly available data, as not all injuries and their subsequent management details could have been disclosed. This predisposes the study to underreporting or misclassification bias. In addition, the different training regimens, fighting styles, and rehabilitation protocols among fighters may introduce different confounding variables that are hard to control for. Finally, the small population size in our study may have limited the statistical power, potentially rendering some observed trends statistically insignificant despite their clinical relevance.

## 5. Conclusions

Shoulder injuries in professional MMA pose a significant challenge to competitive performance, leading to marked declines in winning percentages and KD effectiveness. The high recurrence rate and predominance of labral tears underscore the vulnerability of the shoulder joint in this high-impact sport, reinforcing the need for comprehensive rehabilitation strategies. While surgical interventions and rehabilitation enable a return to competition, post-injury performance deficits suggest existing recovery protocols may be insufficient. To mitigate performance decline and safeguard athlete longevity, future efforts should focus on optimizing rehabilitation frameworks, establishing evidence-based return-to-sport criteria, and strengthening injury prevention measures in MMA.

## Figures and Tables

**Table 1:** Demographic and fighter characteristics.

Characteristic	N (%)
**Organization**	
UFC	25 (92.6%)
Bellator	1 (3.7%)
Strikeforce	1 (3.7%)
**Injury Recurrence**	
No	21 (77.8%)
Yes	6 (22.2%)
**Weight Division**	
Flyweight	4 (14.8%)
Bantamweight	3 (11.1%)
Featherweight	3 (11.1%)
Lightweight	2 (7.4%)
Welterweight	3 (11.1%)
Middleweight	3 (11.1%)
Light Heavyweight	4 (14.8%)
Heavyweight	5 (18.5%)
**Unilateral vs. Bilateral**	
Unilateral	22 (81.5%)
Bilateral	5 (18.5%)

**Table 2:** Characteristics of reported shoulder injuries.

Injury Characteristic	N (%)
**Injury Type**	
Torn Labrum	14 (41.2%)
Torn Rotator Cuff	3 (8.8%)
Torn Biceps/SLAP tear	2 (5.9%)
AC Joint Tear	3 (8.8%)
Torn Labrum + Torn Rotator Cuff	4 (11.7%)
Torn Labrum + Torn Rotator Cuff + Torn Biceps	2 (5.9%)
Unknown	6 (17.6%)
**Required Surgery**	
No	5 (14.7%)
Yes	26 (76.5%)
Unknown	3 (8.8%)
**Setting**	
Training	13 (38.2%)
Fight	15 (44.1%)
Unknown	6 (17.6%)

## Data Availability

The data presented in this study are available on request from the corresponding author (data are not publicly available due to privacy or ethical restrictions).
